# Supplementary material for: The conserved RNA-binding protein Seb1 promotes cotranscriptional ribosomal RNA processing by controlling RNA polymerase I progression
Source: Nat Commun. 2023 May 25;14:3013. doi: 10.1038/s41467-023-38826-6 (PMC10212976; doi:10.1038/s41467-023-38826-6)
Supplement: Supplementary file 1 — Supplementary Information [file 41467_2023_38826_MOESM1_ESM.pdf]

SUPPLEMENTARY INFORMATION

Inventory of Supplementary Information section:

- 5 supplementary Figures (Supplementary Fig. 1-5)
- 2 supplementary Tables (Supplementary Tables 1 and 2)

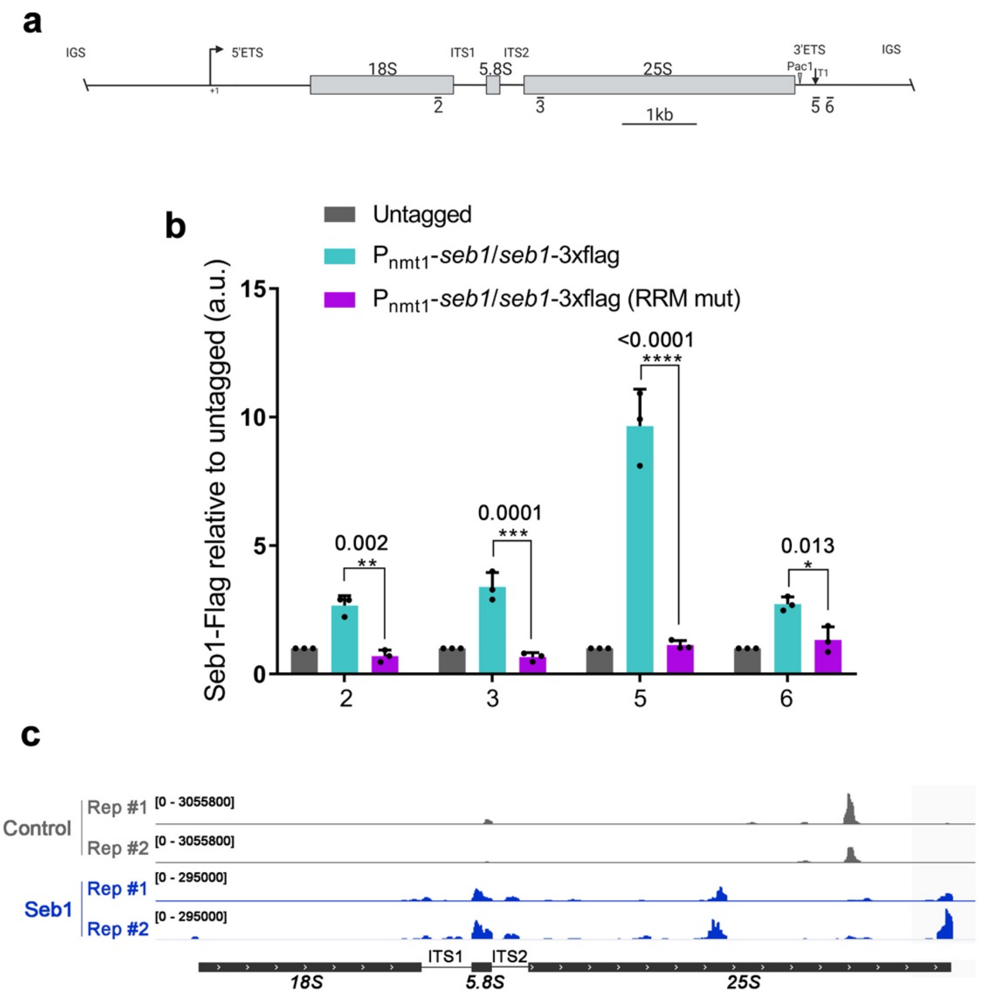

Supplementary Figure 1.
(**a**) Schematic of the rRNA gene. The bars under the rDNA indicate the positions of the PCR products used in the ChIP-qPCR analysis shown in Fig. S1b. (**b**) ChIP analysis of wild-type and RRM mutant (see Fig. 4a for description of substitutions) versions of Seb1-FLAG along the *rDNA*. Control wild-type cells (grey bars) were used as negative control for the anti-FLAG ChIP assays. Data and error bars represent the mean and standard deviation of N=3 independent experiments. *P value <0.05; **P value <0.01; ***P value <0.001 ****P value <0.0001; as determined by unpaired Student's t test corrected for multiple comparisons using the Holm-Sidak method.. (**c**) The number of reads mapping to each nucleotide of the rDNA sequence encoding the 35S pre-rRNA is shown above a schematic view of the rRNA gene. Results from two independent replicates (Rep) using an untagged control strain (grey) and a strain expressing Seb1-HTP (blue) are shown.

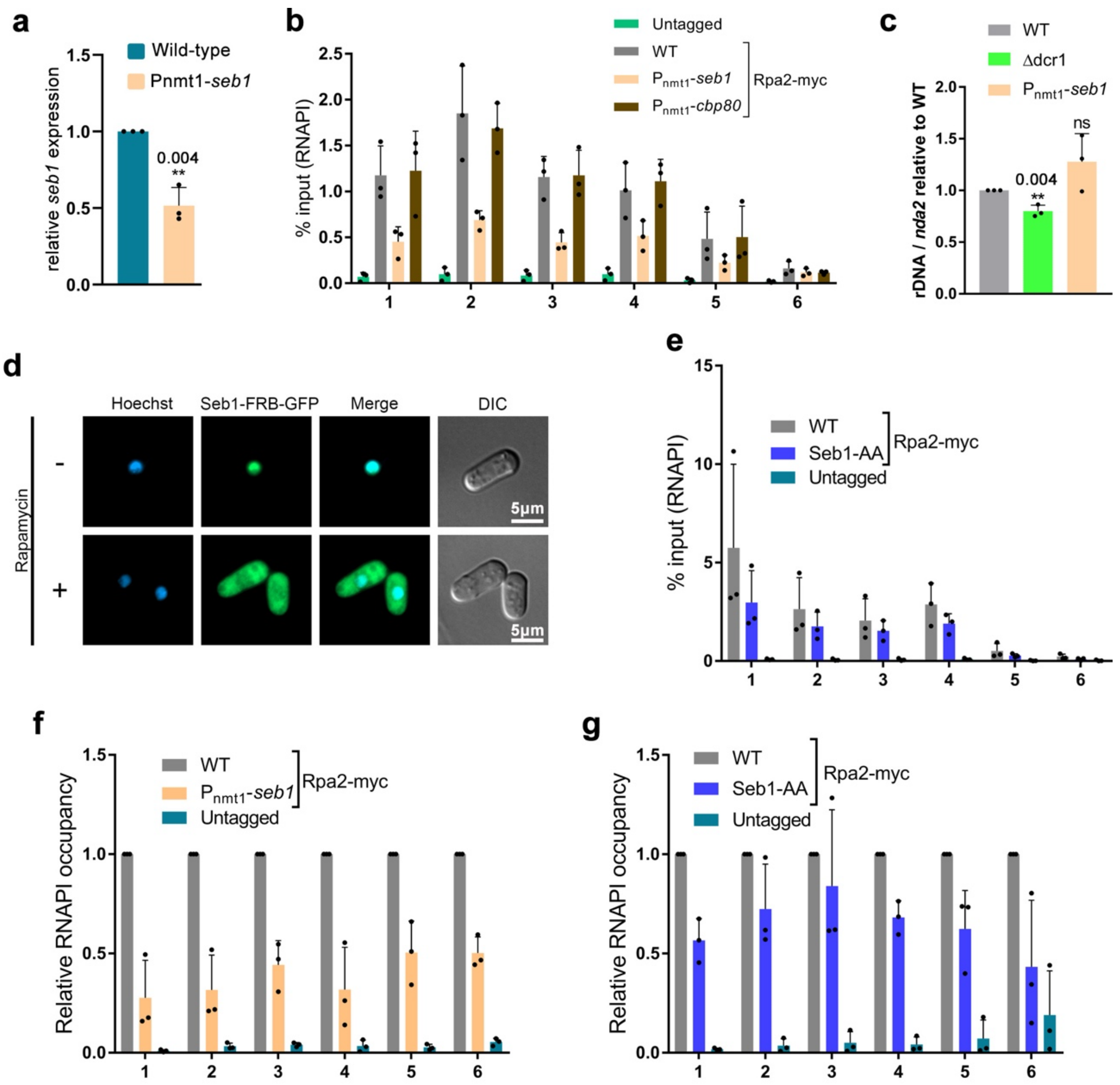

Supplementary Figure 2.

(**a**) RT-qPCR analysis of *seb1* expression using total RNA prepared from the indicated strains 12-14h after the addition of thiamine. RT-qPCR data were normalized to the *nda2* housekeeping mRNA and fold changes expressed relative to the wild-type control strain. Data and error bars represent the mean and standard deviation of N=3 independent experiments. **P value <0.01; as determined by unpaired Student's t test corrected for multiple comparisons using the Holm-Sidak method. (**b**) ChIP-qPCR analysis of RNAPI subunit (Rpa2-myc) in wild-type, *Pnmt1-seb1*, *Pnmt1-cbp80* strains on the rDNA repeats (see Fig. 1c) after the addition of thiamine for 10-12h. Data and error bars represent the mean and standard deviation of N=3 independent experiments. (**c**) rDNA copy number determined by qPCR of genomic DNA from WT, *dcr1* knockout, and *Pnmt1-seb1* cells. rDNA copy number is normalized to *nda2* locus and expressed relative to WT. *dcr1* knockout was previously demonstrated to have reduced rDNA copy number (PMID : 25417108) and was used as a control. Data and error bars represent the mean and standard deviation of N=3 independent experiments. ns P value >0,05; **P value <0.01; as determined by unpaired Student's t test corrected for multiple comparisons using the Holm-Sidak method. (**d**) Representative images of the Seb1 anchor away strain (Seb1-FRB-GFP) natural nuclear localization (top panel) and relocalization to the cytoplasm 2h after rapamycin treatment (bottom panel). The experiment was done 4 times from independent biological replicates. (**e**) ChIP-qPCR analysis of RNAPI subunit (Rpa2-myc) in wild-type and Seb1 anchor away (Seb1-AA) strains on the rDNA repeats after the addition of rapamycin for 2h. Data and error bars represent the mean and standard deviation of N=3 independent experiments. (**f** and **g**) ChIP-qPCR analysis of RNAPI relative occupancy across the rDNA in *Pnmt1-seb1* (**f**) and Seb1-AA (**g**) strains. Data and error bars represent the mean and standard deviation of N=3 independent experiments.

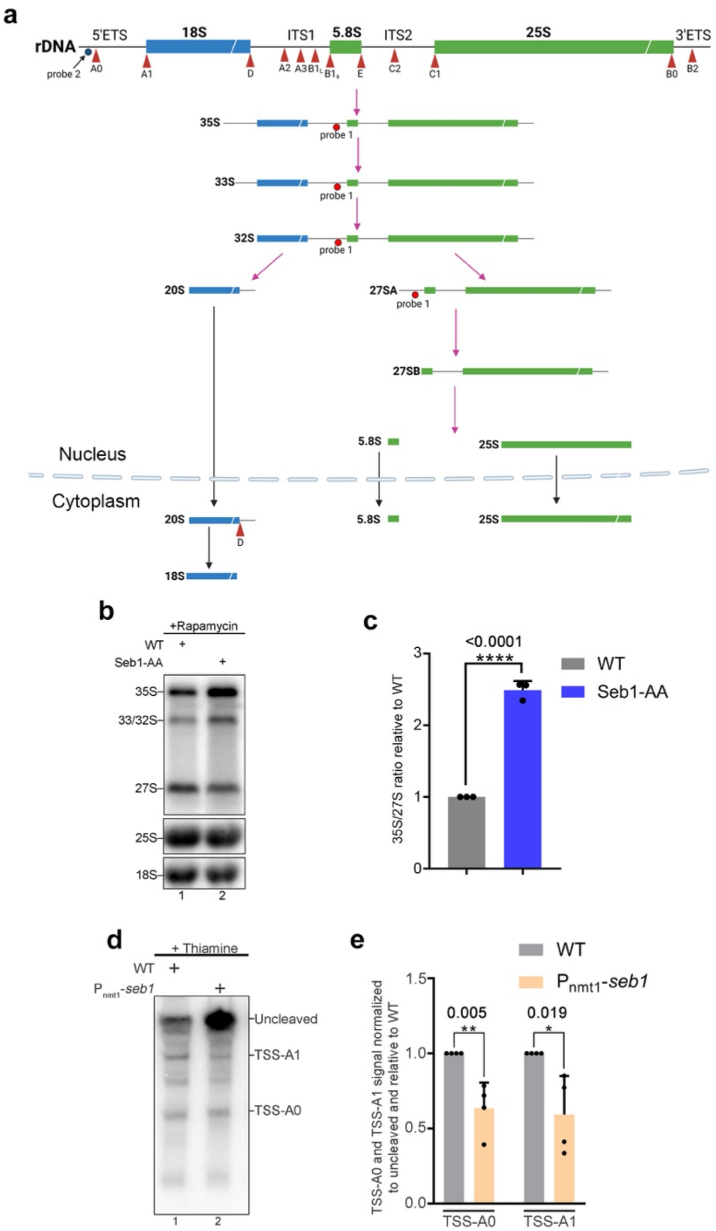

Supplementary Figure 3.

(a) Schematic of major pre-rRNA processing steps in fission yeast. The position of the ITS1-specific (Probe 1; red ball) and 5'-ETS-specific (Probe 2; blue ball) probes used to detect rRNA intermediates by Northern blotting are shown. **(b)** Northern Blot analysis of total RNA prepared from wild-type (lane 1) and Seb1-AA (lane 2) strains following addition of rapamycin for 2h. *Top*, the membrane was analyzed using a probe complementary to ITS1-specific sequences (Probe 1; Fig. S3a). *Middle and bottom*, mature rRNAs were analyzed using probes complementary to 25S- and 18S-specific sequences, respectively. Pre-rRNAs and mature rRNAs are indicated on the left. **(c)** Quantification of 35S/27S pre-rRNA ratio in the Seb1-AA strain relative to the wild-type strain. Data and error bars represent the mean and standard deviation of N=3 independent experiments. *****P* value <0.0001, as determined by unpaired two-tailed Student's *t* test. **(d)** Northern Blot analysis (6% PAGE-Urea gel) of total RNA prepared from wild-type (lane 1) and *Pnmt1-seb1* (lane 2) strains following addition of thiamine for 10-12h. Membranes were analyzed using a probe complementary to 5'-ETS-specific sequences (Probe 2 : Fig. S3a). TSS-A0 and TSS-A1 RNA products as well as uncleaved rRNA precursors are indicated on the right. (**e**) Quantification of TSS-A0 and TSS-A1 signals normalized to uncleaved pre-rRNA in the *Pnmt1-seb1* strain relative to the wild-type strain. Data and error bars represent the mean and standard deviation of 4 independent experiments. **P* value <0.05; ***P* value <0.01, as determined by unpaired two-tailed Student's *t* tests corrected for multiple comparisons using the Holm-Sidak method.

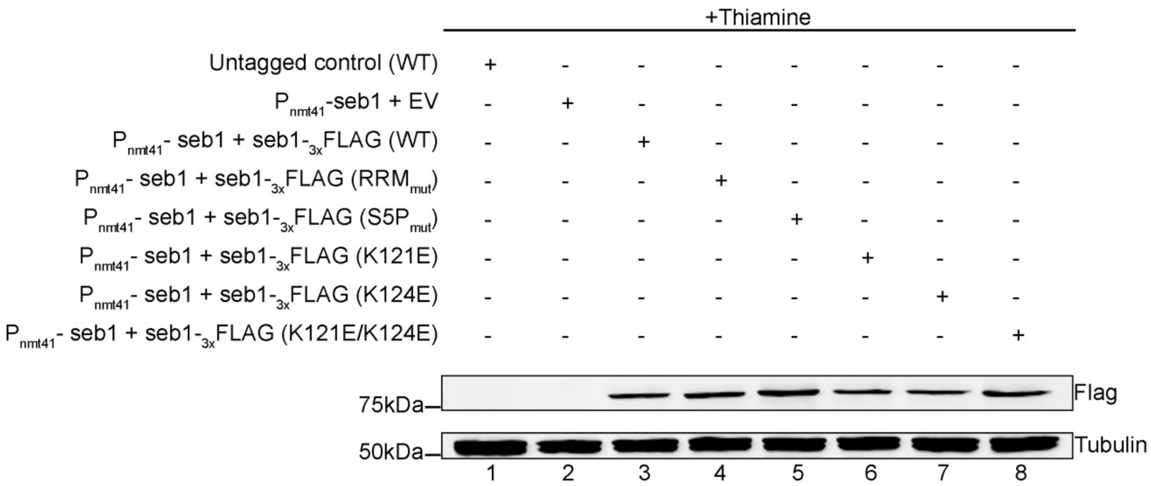

Supplementary Figure 4.
Western blot analysis of Seb1-Flag from the indicated strains after thiamine-dependent depletion of endogenous Seb1. The experiment was done N=3 independent biological replicates.

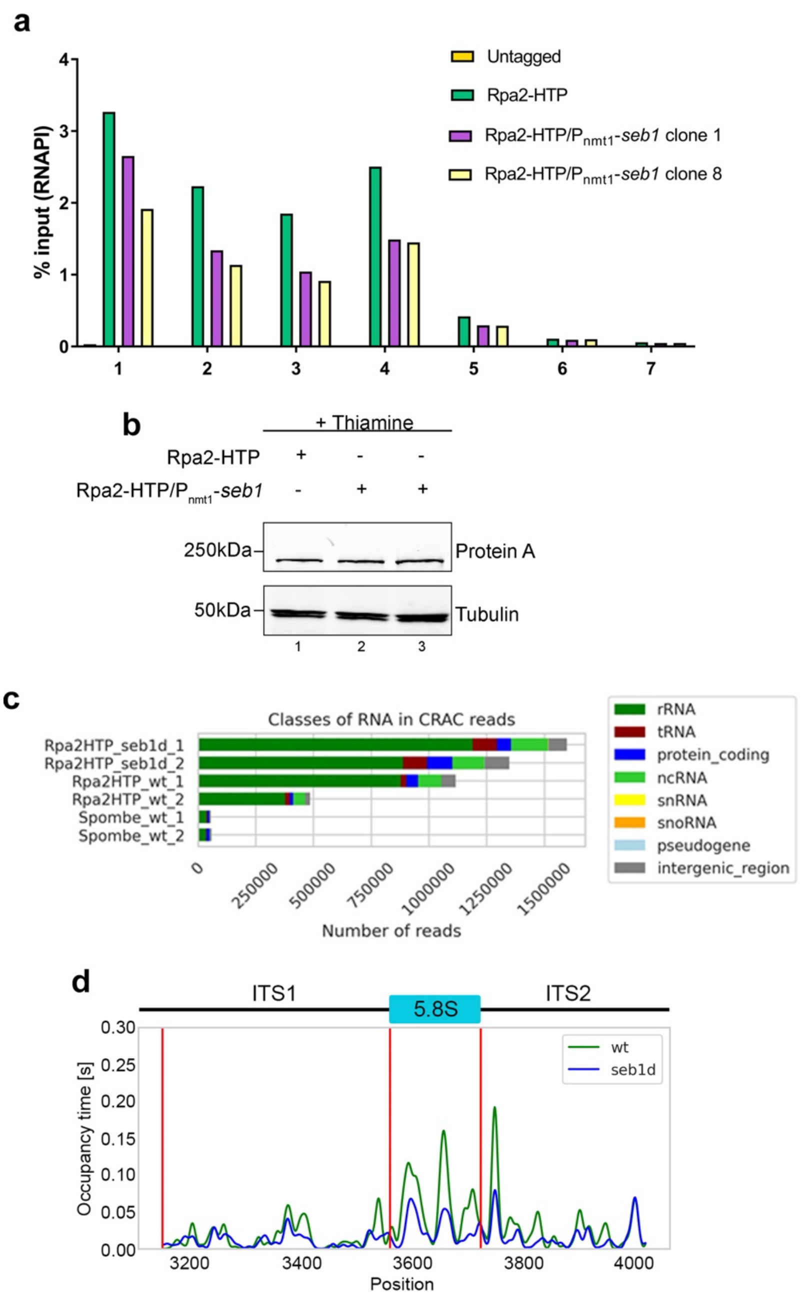

Supplementary Figure 5.

(**a**) ChIP-qPCR analysis of RNAPI subunit (Rpa2-HTP) in wild-type and two independent clones with the *Pnmt1-seb1* allele on the rDNA repeats after the addition of thiamine for 10-12h. Clone 1 was used for the RNAPI CRAC assays shown in Fig. 5. **(b)** Western blot analysis of Rpa2-HTP in wild-type (lane 1), *Pnmt1-seb1* clone 1 (lane 2), and *Pnmt1-seb1* clone 8 (lane 3) strains after the addition of thiamine. ProA- and Tubulin-specific antibodies were used to detect Rpa2-HTP and Tubulin, respectively. The experiment was done N=2 independent biological replicates. **(c)** Comparison of the distribution of CRAC reads across the indicated categories of genes for Rpa2 in wild-type (Rpa2HTP_wt) and Seb1-depleted (Rpa2HTP_seb1d) cells from two independent replicates. Classes of CRAC reads from the untagged control strain (Spombe_wt) are also shown. **(d)** Rpa2 CRAC profiles across the ITS1, 5.8S, and ITS2 regions, presented as occupancy time (in seconds) for wild-type (green) and *Pnmt1-seb1* (blue) cells after the addition of thiamine for 10-12h. The solid lines mark the average for two biological replicates.

Supplementary Table 1. List of strains used in this study.

Name	ID	Reference	Genotype
WT ChIP	FBY13	PMID: 17213188	h+ S. pombe: ade6M210 leu1-32 ura4-D18 his3-D1
WT mutants	FBY106	PMID: 17213188	h+ ade6-M216 leu1-32 ura4-D18 his3-D1
WT Anchor away	FBY2066	PMID: 24733494	h- torSE::KanMX6 fkh1::URA+ leu1-32::(nmt1-rpl13-2FKBP12-leu1+) ura4-D18 leu1-32 ade6-M210
Rpa2-myc (AA control)	FBY2603	PMID: 34352089	h- torSE::KanMX6 fkh1::URA+ leu1-32::(nmt1-rpl13-2FKBP12-leu1+) rpa2-13myc-HphR ura4-D18 leu1-32 ade6-M210
Seb1-AA/Rpa2-myc	FBY2667	This study	h- torSE::KanMX6 fkh1::URA+ leu1-32::(nmt1-rpl13-2FKBP12-leu1+) ura4-D18 leu1-32 ade6-M210 Seb1-FRB-GFP::natMX6
Pnmt1-seb1/Rpa2-myc	FBY2645	This study	h+ ade6-M21? leu1-32 ura4-D18 his3-D1 KanMX6::P41nmt1-seb1 rpa2-13myc-NatR
Rpa2-myc (ChIP control)	FBY2646	This study	h+ ade6-M21? leu1-32 ura4-D18 his3-D1 rpa2-13myc-NatR
Seb1-TAP	FBY885	PMID: 27401558	h? ade6-M21? leu1-32 ura4-D18 his3-D1 Seb1-TAP::KanMX6
WT/Ura-	FBY1712	This study	h+ his7-366 leu1-32 ura4-d18 ade6-m210 pFB45::URA4
Pnmt1-seb1/Ura-	FBY2063	This study	h+ his7-366 leu1-32 ura4-d18 ade6-m210 pFB45::URA4 natMX6::p41nmt1-seb1
Pnmt1-seb1/EV	FBY1561	PMID: 27401558	h+ ade6-M21? leu1-32 ura4-D18 his3-D1 P41NMT1-SEB1::KANMX6 pFB366::ADE6
Pnmt1-seb1/Seb1-3xFlag	FBY1829	PMID: 27401558	h+ ade6-M? leu1-32 ura4-D18 his3-D1 P41nmt1-seb1::kanMX6 pFB943::ade6
Pnmt1-seb1/Seb1-3xFlag RRMmut	FBY1833	PMID: 27401558	h+ ade6-M? leu1-32 ura4-D18 his3-D1 P41nmt1-seb1::kanMX6 pFB946::ade6
Pnmt1-seb1/Seb1-3xFlag S5mut	FBY1920	PMID: 27401558	h+ ade6-M? leu1-32 ura4-D18 his3-D1 P41nmt1-seb1::kanMX6 pFB1037::ade6
Pnmt1-seb1/Seb1-3xFlag K124E	FBY2754	This study	h+ ade6-M? leu1-32 ura4-D18 his3-D1 P41nmt1-seb1::kanMX6 pFB1503::ade6
Pnmt1-seb1/Seb1-3xFlag K121E/K124E	FBY2756	This study	h+ ade6-M? leu1-32 ura4-D18 his3-D1 P41nmt1-seb1::kanMX6 pFB1507::ade6
Pnmt1-seb1/Seb1-3xFlag K121E	FBY2769	This study	h+ ade6-M? leu1-32 ura4-D18 his3-D1 P41nmt1-seb1::kanMX6 pFB1505::ade6
Rpa2-HTP (CRAC control)	FBY2718	This study	h+ his7-366 leu1-32 ura4-d18 ade6-m210 pFB45::URA4 Rpa2-HTP::KanMx6
Pnmt1-seb1/Rpa2-HTP	FBY2728	This study	h+ his7-366 leu1-32 ura4-d18 ade6-m210 pFB45::URA4 Rpa2-HTP::KanMx6 P41nmt1-seb1::natMx6
Rrn3-myc (ChIP control)	FBY2766	This study	h- S. pombe: ade6M210 leu1-32 ura4-D18 his3-D1 Rrn3-13myc::natMX6
Pnmt1-seb1/Rrn3-myc	FBY2770	This study	h? ade6-M21? leu1-32 ura4-D18 his3-D1 D1 Rrn3-13myc::natMX6 KanMX6::P41nmt1-seb1
Seb1-BirA-myc	FBY2683	This study	h+ S. pombe: ade6M210 leu1-32 ura4-D18 his3-D1 Seb1-TurboID-3MYC::kanMX6
Seb1-HTP	FBY1858	PMID: 27401558	h+ ade6-M216 leu1-32 ura4-D18 his3-D1 Seb1-HTP::kanMX6
Pnmt1-cbp80/Rpa2-myc	FBY2926	This study	h+ ade6-M21? leu1-32 ura4-D18 his3-D1 KanMX6::P81nmt1-cbp80 rpa2-13myc-NatR

Supplementary Table 2. List of oligonucleotides used in this study.

Target	Number	Orientation	Sequence (5'-3')	Used in
rRNA NTS-0 (-1200 of TSS)	FB6295	F	ggctggaatgcaaaaacagt	figure 1
rRNA NTS-0 (-1200 of TSS)	FB6296	R	cttttgacccatttgcctgt	figure 1
rRNA 5'ETS -1	FB6033	F	ggtaccttgataccgcaagg	figure 1, 2, S2, S5
rRNA 5'ETS-1	FB6034	R	gattctccagcaggaaaagag	figure 1, 2, S2, S5
rRNA promoter-A (-416 of TSS)	FB6632	F	tcagagatgggtgtgggaag	figure 2
rRNA promoter-A (-416 of TSS)	FB6633	R	ttgtttgttgaaaccccttttt	figure 2
rRNA promoter-B (-156 of TSS)	FB6289	F	tgtggtggatagttttctggtg	figure 2
rRNA promoter-B (-156 of TSS)	Fb6290	R	tcctttcaaccaccactcct	figure 2
rRNA promoter-C (+211 of TSS)	FB6630	F	ttctctttgtgttggcatcg	figure 2
rRNA promoter-C (+211 of TSS)	FB6631	R	acggaccttcttttcattcttc	figure 2, S3
rRNA 18S-2	FB723	F	cagcttgcgttgaatacgtccc	figure 1, 2, S2, S5
rRNA 18S-2 and NB	FB724	R	agccaatccagaggcctcacta	figure 1, 2, 3, 4, S2, S3, S5
rRNA 25S-3	FB6169	F	ccgtctttggtcgattggat	figure 1, 2, S2, S5
rRNA 25S-3	FB6170	R	cggtctctcgccaatatttagc	figure 1, 2, S2, S5
rRNA 25S-4	FB6863	F	ctcgaaatttgaggacagag	figure 1, 2, S2, S5
rRNA 25S-4	FB6864	R	tcgaagaatcaaaaagcaac	figure 1, 2, S2, S5
rRNA 3'ETS-5 (+400)	FB6037	F	gtattagtattaggaattgg	figure 1, 2, S2, S5
rRNA 3'ETS-5 (+400)	FB6038	R	tactaggatttgtgcattacc	figure 1, 2, S2, S5
rRNA 3'ETS-6 (+600)	FB6137	F	agtggtaaggtaggtcgtga	figure 1, 2, S2, S5
rRNA 3'ETS-6 (+600)	FB6138	R	tgcactgcgttaaatccctc	figure 1, 2, S2, S5
rRNA 3'ETS-7 (+775)	FB6139	F	cacacacacacacacgctaa	figure 1
rRNA 3'ETS-7 (+775)	FB6140	R	acacgcttggtttgtttgtaa	figure 1
rRNA A2/A3 NB probe in ITS1	Fb111	R	tgcttggcatgcaacaa	figure 3, 4, S3
rRNA 25S NB	FB728	R	ttctggcacggattctggcttaga	figure 3, 4, S3
nda2	FB202	F	gctatgagccaccccaacac	figure S2
nda2	FB203	R	agaccaagcttcagcaatcga	figure S2
