# Supplementary material for: The conserved RNA-binding protein Seb1 promotes cotranscriptional ribosomal RNA processing by controlling RNA polymerase I progression
Source: Nat Commun. 2023 May 25;14:3013. doi: 10.1038/s41467-023-38826-6 (PMC10212976; doi:10.1038/s41467-023-38826-6)
Supplement: Supplementary file 3 — Description of Additional Supplementary Files [file 41467_2023_38826_MOESM3_ESM.pdf]

Description of Additional Supplementary Files

File Name: Supplementary Data 1
Description: List of high confidence proteins identified by proximity-dependent biotinylation assay of Seb1-TurboID. Mass spectrometry data are for the control (FBY13) and Seb1-TurboID (FBY2683) strains.

File Name: Supplementary Data 2
Description: List of 268 common proteins identified by Affinity Purification-mass spectrometry of Seb1-TAP and Proximity-Dependent Biotinylation-mass spectrometry of Seb1-TurboID with a minimum of 2 unique peptides and a minimum of 5% amino acid sequence coverage.
