# Supplementary material for: The conserved RNA-binding protein Seb1 promotes cotranscriptional ribosomal RNA processing by controlling RNA polymerase I progression
Source: Nat Commun. 2023 May 25;14:3013. doi: 10.1038/s41467-023-38826-6 (PMC10212976; doi:10.1038/s41467-023-38826-6)
Supplement: Supplementary file 6 — Reporting Summary [file 41467_2023_38826_MOESM6_ESM.pdf]

nature portfolio

Corresponding author(s): Francois Bachand

Last updated by author(s): May 1, 2023

Reporting Summary

Nature Portfolio wishes to improve the reproducibility of the work that we publish. This form provides structure for consistency and transparency in reporting. For further information on Nature Portfolio policies, see our Editorial Policies and the Editorial Policy Checklist.

Statistics

For all statistical analyses, confirm that the following items are present in the figure legend, table legend, main text, or Methods section.

n/a	Confirmed	
☐	☒	The exact sample size (*n*) for each experimental group/condition, given as a discrete number and unit of measurement
☐	☒	A statement on whether measurements were taken from distinct samples or whether the same sample was measured repeatedly
☐	☒	The statistical test(s) used AND whether they are one- or two-sided<br>*Only common tests should be described solely by name; describe more complex techniques in the Methods section.*
☒	☐	A description of all covariates tested
☒	☐	A description of any assumptions or corrections, such as tests of normality and adjustment for multiple comparisons
☐	☒	A full description of the statistical parameters including central tendency (e.g. means) or other basic estimates (e.g. regression coefficient) AND variation (e.g. standard deviation) or associated estimates of uncertainty (e.g. confidence intervals)
☐	☒	For null hypothesis testing, the test statistic (e.g. *F*, *t*, *r*) with confidence intervals, effect sizes, degrees of freedom and *P* value noted<br>*Give P values as exact values whenever suitable.*
☒	☐	For Bayesian analysis, information on the choice of priors and Markov chain Monte Carlo settings
☒	☐	For hierarchical and complex designs, identification of the appropriate level for tests and full reporting of outcomes
☒	☐	Estimates of effect sizes (e.g. Cohen's *d*, Pearson's *r*), indicating how they were calculated

Our web collection on statistics for biologists contains articles on many of the points above.

Software and code

Policy information about availability of computer code

Data collection	No software was used for data collection.
Data analysis	CRAC analysis and visualization were performed in Python using pandas, matplotlib and trxtools which are available on GitHub (https://github.com/TurowskiLab/trxtools).<br>For Mass spectrometry analyses, Xcalibur (version 4.1) and MaxQuant (version 1.6.2.2) were used, which are open source.

For manuscripts utilizing custom algorithms or software that are central to the research but not yet described in published literature, software must be made available to editors and reviewers. We strongly encourage code deposition in a community repository (e.g. GitHub). See the Nature Portfolio guidelines for submitting code & software for further information.

Data

Policy information about availability of data

All manuscripts must include a data availability statement. This statement should provide the following information, where applicable:

- Accession codes, unique identifiers, or web links for publicly available datasets
- A description of any restrictions on data availability
- For clinical datasets or third party data, please ensure that the statement adheres to our policy

The data from the Rpa2-HTP CRAC analysis can be accessed through Gene Expression Omnibus accession codes GSE212930. The mass spectrometry proteomics data was deposited to the ProteomeXchange Consortium via the PRIDE partner repository with the data set identifier PXD037768.

Human research participants

Policy information about studies involving human research participants and Sex and Gender in Research.

Reporting on sex and gender	not applicable
Population characteristics	not applicable
Recruitment	not applicable
Ethics oversight	not applicable

Note that full information on the approval of the study protocol must also be provided in the manuscript.

Field-specific reporting

Please select the one below that is the best fit for your research. If you are not sure, read the appropriate sections before making your selection.

☒ Life sciences ☐ Behavioural & social sciences ☐ Ecological, evolutionary & environmental sciences

For a reference copy of the document with all sections, see nature.com/documents/nr-reporting-summary-flat.pdf

Life sciences study design

All studies must disclose on these points even when the disclosure is negative.

Sample size	Cell samples were collected from 50- and 100-ml of mid-log phase cultures of Scizosaccharomyces pombe. These sample sizes for these types of experiments are common in the yeast field.
Data exclusions	No data were excluded from analyses.
Replication	All of the replicates performed in this study were "biological replicates from independent cell cultures" and the number of biological replicate performed for each experiment is specified in the legend of each figure.
Randomization	No randomization was required because we did not have large datasets or trials.
Blinding	Blinding was not required for the type of experiments we present.

Reporting for specific materials, systems and methods

We require information from authors about some types of materials, experimental systems and methods used in many studies. Here, indicate whether each material, system or method listed is relevant to your study. If you are not sure if a list item applies to your research, read the appropriate section before selecting a response.

Materials & experimental systems

n/a	Involved in the study	
☐	☒	Antibodies
☒	☐	Eukaryotic cell lines
☒	☐	Palaeontology and archaeology
☒	☐	Animals and other organisms
☒	☐	Clinical data
☒	☐	Dual use research of concern

Methods

n/a	Involved in the study	
☐	☒	ChIP-seq
☒	☐	Flow cytometry
☒	☐	MRI-based neuroimaging

Antibodies

Antibodies used	Mouse monoclonal anti-myc (9E10, sc-40, 1:500 (v/v) dilution), anti-Flag (Sigma-Aldrich, F1804; 1:500 (v/v) dilution) , and rabbit polyclonal antibody against protein A (Sigma-Aldrich, P3775; 1:10,000 (v/v) dilution) in the case of HTP-tagged proteins. Membranes were probes with goat anti-mouse secondary antibodies coupled to AlexaFluor 680 (Life Technologies, A-21057; 1:15000 (v/v) dilution). A mouse monoclonal antibody specific to α-tubulin (Sigma-Aldrich, T5168; 1:1000 (v/v) dilution).
Validation	Antibodies were validate using appropriate S. pombe strains that had been treated under appropriate conditions or were deleted for

Validation	an appropriate gene, compared to untreated/wild-type controls. Anti-myc recognized single 160-kDa and 85-kDa bands from lysates of cells expressing Rpa2-myc and Rrn3-myc, but not in control untagged cells. Anti-tubulin recognized a doublet of the expected molecular weight of 55-kDa. Anti-Flag recognized single 85-kDa bands from lysates of cells expressing Seb1-Flag, but not in control untagged cells.

ChIP-seq

Data deposition

☒ Confirm that both raw and final processed data have been deposited in a public database such as GEO.

☒ Confirm that you have deposited or provided access to graph files (e.g. BED files) for the called peaks.

Data access links<br>*May remain private before publication.*	-The data from the Rpa2-HTP CRAC analysis can be accessed through Gene Expression Omnibus accession codes GSE212930.<br>-The following secure token has been created to allow review of record GSE212930 while it remains in private status: ubutayywvjovtcb<br>-Additional ChIP-seq (Fig. 1A) and CRAC (Fig. 1G-1H; seb1-HTP) data have been previously published and accession numbers to raw and final processed data are available in the cited studies.
Files in database submission	GSM6562873 Rpa2-HTP WT_1<br>GSM6562874 Rpa2-HTP WT_2<br>GSM6562876 Rpa2-HTP seb1d_1<br>GSM6562877 Rpa2-HTP seb1d_2<br>GSM6562878 untagged_1<br>GSM6562879 untagged_2
Genome browser session<br>(e.g. UCSC)	No longer available.

Methodology

Replicates	CRAC analysis of Rpa2-HTP in wild-type and Seb1-depleted cells was performed in two independent biological replicates.
Sequencing depth	Worked only with the reads that contain the 3' linker. For BigWig used both: all reads or just the 3' end position of each read.<br>EP211100_Rpa2HTP_seb1d_1 : 567630 reads<br>EP211100_Rpa2HTP_wt_1 : 431856 reads<br>EP211100_Rpa2HTP_wt_2: 179272 reads<br>EP211100_Rpa2HTP_seb1d_2: 425072 reads
Antibodies	IgG Dynabeads (Life Technologies, 11041)
Peak calling parameters	No peak calling tools were used in this study.
Data quality	Illumina sequencing data were demultiplexed using in-line barcodes and in this form were submitted to GEO. First quality control step was performed using FastQC software (http://www.bioinformatics.babraham.ac.uk/projects/fastqc/) considering specificity of CRAC data.
Software	CRAC analysis and visualization were performed in Python using pandas, matplotlib and trxtools which are available on GitHub (https://github.com/TurowskiLab/trxtools).
